# Drying Model and Mechanism of Sugar Beet Pulp Based on Its Crosslinking with Ca^2+^ and Cu^2+^

**DOI:** 10.3390/foods14193362

**Published:** 2025-09-28

**Authors:** Guili Jiang, Yanxia Zhang, Donghui Luo, Siming Zhu, Yutao Wang, Wanzhi Li

**Affiliations:** 1 College of Food Science and Engineering, Guangdong Ocean University, Yangjiang 529500, China; jgl@gdou.edu.cn; 2 College of Life and Geography, Kashi University, Kashi 844000, China; 3 School of Food Science and Engineering, South China University of Technology, Guangzhou 510641, China; 4 Key Laboratory of Biological Resources and Ecology of Pamirs Plateau in Xinjiang Uygur Autonomous Region, Kashi University, Kashi 844000, China; 5 Institute of Food and Health, Yangtze Delta Region Institute of Tsinghua University Zhejiang, Jiaxing 314006, China; 6 Xinjiang Lvxiang Sugar Co., Ltd., Tacheng 834700, China

**Keywords:** sugar beet pulp, drying kinetic study, mathematical drying model, dryness–strengthening mechanism, crosslinking with metal ions

## Abstract

Sugar beet pulp (SBP) is a by-product from the sugar industry with low value. As a feed, SBP needs to be dried. However, the drying process takes too much energy, leading to potential environmental issues caused by coal use. This paper raised and tried a crosslinking method to shorten the drying process, save energy consumption, and increase the value of SBP. This paper aimed to reduce the water-holding ability of SBP while obtaining animal feed with higher nutritional value. First, the crosslinking method was used to evaluate its dryness–strengthening effect. Second, three factors were evaluated: operating temperature, solution pH, and cationic concentration. Third, a kinetic study was performed on the drying process of SBP through its crosslinking with macro-elements (Ca^2+^, Cu^2+^) using drying models; the characterization of Ca^2+^-SBP and Cu^2+^-SBP using FTIR, SEM, and XRD; and possible drying mechanisms, which were discussed using an egg box model and a simple quantum chemical calculation. Results showed that the dryness–strengthening and value-adding idea is more practical through a Ca^2+^-crosslinking method, rather than through crosslinking with Cu^2+^. Under experimental conditions, wet SBP with 2 g of dry base reacts to Ca^2+^ under optimized conditions of 1000 mg/L Ca^2+^ solution at pH 6.0 and 40 °C for 135 min, with a moisture content of 5.23 g/g as a water-holding index. Compared with SBP, the moisture content of the crosslinking SBP on a dry basis was reduced by ~30–40%. The Midilli–Kucuk model was the most suitable model to describe the hot-air drying process of SBP, while Ca^2+^ or Cu^2+^ can crosslink to the galacturonic acid in pectin and form an “egg-box” model. SBP binds with Ca^2+^ or Cu^2+^ through its carboxyl groups, as testified by a combination analysis of FTIR, SEM, and XRD. As a result, the SBP dried through the Ca^2+^-crosslinking or Cu^2+^-crosslinking method can be directly used as a feed additive with good economic benefit and without the post-treatment problem as a bio-sorbent.

## 1. Introduction

Worldwide, the output of beet sugar was about 36.86 million tons in the producing season of 2024/25, meaning that about 265 million tons of sugar beet pulp was produced. China is the second-largest consumer country and the fourth-largest sugar-producing country. Beet sugar accounts for approximately 10 percent of the sugar yield in China. Sugar beet pulp is a waste from the beet sugar industry. As the largest beet sugar base in China, Xinjiang accounts for about 60% of the total beet sugar yield in China. Each year, the Chinese beet sugar industry produces more than 10 million tons of SBP waste [1]. Usually, processing 1 t of sugar beet root yields about 0.9 t of SBP. So, SBP is high in yield and low in cost. SBP has a wide range of application potential in the fields of biofuels, animal feed, food processing, the pharmaceutical industry, and effluent treatment as a bio-sorbent [2,3].

Xinjiang, located in central Eurasia and on the northwest border of China, is a typical arid region. The hardness (1027 mg/L, similar to local tap water or groundwater hardness) of its surface water is much higher than that of the Chinese national standard (450 mg/L) (GB 5749-2022) [4], which poses a serious danger to people’s health or organisms. SBP can be used as a bio-sorbent; it can effectively absorb Ca^2+^ and Mg^2+^ in hard water [5]. Xinjiang is rich in mineral resources and cotton resources, and the processing of these resources generates heavy metal wastewater and dye wastewater, resulting in environmental pollution. The use of SBP as a bio-sorbent can effectively remove pollutants from wastewater, because SBP is rich in carboxyl groups and hydroxyl groups [6,7,8]. As a bio-sorbent, SBP has the advantages of economy, environmental protection, energy conservation, high efficiency, and selectivity. In addition, in subsequent processing, metal ions can be desorbed from SBP by some eluent agents, and SBP can be reused after its desorption [9]. However, elution or regeneration may produce a certain amount of wastewater, varying in quantity.

SBP is mainly used as a feed. For long-distance transportation, water content in SBP is generally reduced by mechanical pressing and hot-air drying or spray drying. However, this drying method is energy-intensive, accounting for approximately 30% of the total energy consumption in beet sugar processing [10]. The high water content of raw SBP accounts for more than 90% of sugar beet, and its high water-binding property is related to the high water content of pectin and binding polysaccharides. The pectin content in SBP is about 24%. Pectin, as a kind of polysaccharide rich in galacturonic acid, plays a vital role in adsorption and binding water in the beetroot tissue, and its main component is D-galacturonic acid, which is linked via the alpha-(1,4)-glycosidic bond. Second, cellulose contains a lot of hydrophilic groups, which swell when exposed to water. Therefore, it is very necessary to seek an efficient and environmentally friendly method for SBP drying.

**Figure 1:**
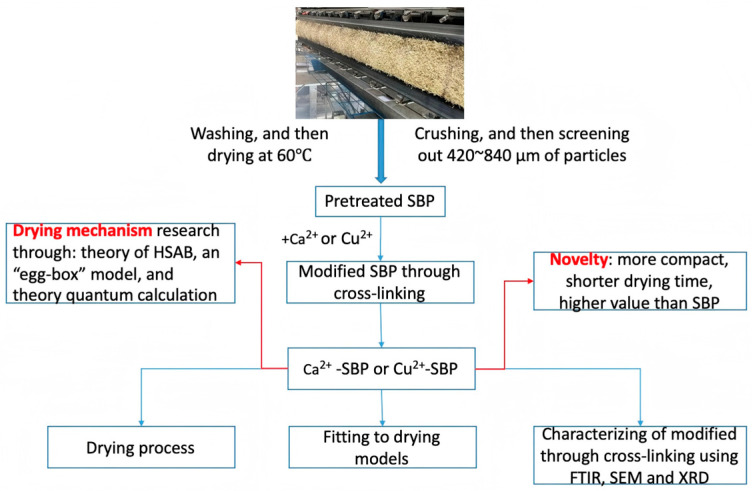
Schematic diagram of an energy-saving drying method based on a crosslinking reaction of SBP with metal ions.

SBP is used as a feed or a bio-sorbent. To overcome the low-value shortcoming of SBP as a bio-sorbent with the post-treatment problem of the complex of SBP toxic adsorbates, to overcome the large energy-consuming shortcoming problem as feed, and to reduce drying energy consumption, SBP can add value or nutrition as a feed, and it is also possible to shorten the drying time. In this paper, a method is presented and practiced, as shown in Figure 1. First, to improve the drying process and the nutritional value of SBP through a crosslinking reaction, SBP was reacted with cationic ions like Ca^2+^ or Cu^2+^ due to its good crosslinking ability. Ca^2+^ can be directly used as a feed additive to improve the nutritional value of feed. Usually, organic calcium has better bioavailability, absorption, and utilization [11]. For example, the addition of Ca^2+^ is very important, especially for dairy cows, which need 70~100 g of calcium per day; this amount of calcium is not enough to be ingested only from feed [12]. SBP contains a large amount of galacturonic acid, which can bind with Ca^2+^ to form calcium galacturonate, which is more easily absorbed by animals and has higher nutritional value than inorganic calcium. At the same time, the crosslinking process strengthens the firmness and hardness of SBP, while decreasing its water-holding ability. It is hoped that this will result in a shorter drying time and faster drying velocity. Second, the effect of temperature and the crosslinking treatment with Ca^2+^ and Cu^2+^ on the moisture content, drying curve, and drying rate of the SBP was studied. In addition, different mathematical drying models were fitted, and a possible drying mechanism was also discussed.

## 2. Materials and Methods

### 2.1. Materials and Reagents

Sugar beet pulp (SBP) was provided from Kashi Aodu Sugar Industry Co., Ltd. (Kashi, China). Anhydrous calcium chloride (CaCl_2_) and anhydrous cupric chloride (CuCl_2_) were obtained from Tianjin Yongsheng Fine Chemical Co., Ltd. (Tianjin, China). All the chemicals were of analytical grade.

### 2.2. Modification of SBP

SBP was washed with distilled water to remove impurities and then dried in an Electric thermostatic drying oven (Shanghai Qixin Technology Co., Ltd., Shanghai, China) at 60 °C to a constant weight. It was subsequently crushed with a pulverizer and screened for uniform particles in the size range of 420 μm~840 μm, then finally stored in a sealed container.

Subsequently, pre-treated SBP was used as the raw material and subjected to a crosslinking reaction with Ca^2+^ or Cu^2+^. As a result, pre-treated SBP, Ca^2+^-SBP, and Cu^2+^-SBP were stored for the following drying experiment. The specific operating process is shown in Figure 1: 2.0 g of pre-treated SBP was immersed in 100 mL of distilled water, 100 mL of 1.0 g/L CaCl_2_, and 100 mL of 1.0 g/L CuCl_2_, respectively. The mixture was stirred in a 50 °C water bath for 60 min; then filtered to remove water; weighed; and dried in hot air at 60 °C, 70 °C, and 80 °C, respectively. The drying time of each sample was recorded, and the effects of metal cation crosslinking on the drying of SBP were analyzed.

### 2.3. Drying Experiment

#### 2.3.1. Effect of Ca^2+^ Concentration and Cu^2+^ Concentration on the SBP-Drying Process Through a Crosslinking Modification

Ten SBP samples of 2 g were sampled and added into a CaCl_2_ solution of 250, 500, 750, 1000, and 1250 mg/L, respectively, and also added into a CuCl_2_ solution of 250, 500, 750, 1000, and 1250 mg/L, respectively. The crosslinking process was performed at natural pH in a 50 °C water bath for 30 min. Then, Ca^2+^-SBP and Cu^2+^-SBP were removed, and the potential moisture of each sample was removed. The two samples were placed in a hot air drying oven at 60 °C and were weighed every 15 min till a constant weight.

#### 2.3.2. Effect of Water Bath Temperatures on the SBP-Drying Process with a Crosslinking Modification

Ten samples of 2 g SBP were sampled, put in 5 flasks containing 100 mL of 1000 mg/L CaCl_2_ in each container, or put in 5 flasks containing 100 mL of 750 mg/L CuCl_2_ in each container. Each flask was put in a water bath for 30 min as a crosslinking reaction. The crosslinking was performed at pH 6.0, and the reaction temperature was set at 40, 50, 60, 70, and 80 °C, respectively. Then, 10 samples were taken out and dried to a constant weight according to Section 2.3.1.

#### 2.3.3. Effect of pH Value on the SBP-Drying Process Through a Crosslinking Modification

Ten samples of 2 g of SBP were sampled, put in 5 flasks containing 100 mL of 1000 mg/L CaCl_2_ in each container, or put in 5 flasks containing 100 mL of 750 mg/L CuCl_2_ in each container. Each flask was placed in a 40 °C water bath for 30 min to initiate the crosslinking reaction. The crosslinking was performed at pH 3.0, pH 4.0, pH 5.0, pH 6.0, and pH 7.0, respectively. Then, 10 samples were taken out and dried to a constant weight according to Section 2.3.1.

### 2.4. Parameter Calculation Method

#### 2.4.1. Moisture Content of Drying Base

Moisture content of the drying base is calculated as follows:

(1) $$M_{t}=\frac{W_{t}-G}{G}$$

where *M_t_* is the dry base moisture content of the material in g/g, *W_t_* is the material mass at time *t* in g, and *G* is the material mass in the drying balance in g.

#### 2.4.2. Ratio of Moisture

The ratio of moisture is calculated as follows:

(2) $$M_{r}=\frac{M_{t}-M_{e}}{M_{0}-M_{e}}$$

where *M_t_* is the dry base moisture content of the material at time *t* in g/g, *M_e_* is the dry base moisture content when the material is in drying equilibrium in g/g, and *M*_0_ is the initial dry base moisture content of the material in g/g. Compared with the initial moisture content *M*_0_ and the moisture content *M_t_* at time *t*, the equilibrium moisture content *M_e_* can be ignored under the general drying condition, so Equation (2) can be simplified as *M_r_* = *M_t_*/*M*_0_.

#### 2.4.3. Drying Rate

The ratio of moisture is calculated as follows:

(3) $$v_{t}=\frac{M_{t}-M_{t-1}}{t_{d}}$$

where *v_t_* is the drying rate in g/(g·min), *M*_*t* − 1_ is the dry base moisture content of the material at time *t* − 1 in g/g, and *t_d_* is the interval between *t* and *t* − 1 in min.

### 2.5. Theoretical Analysis Using Theory Chemical Calculation

The basic structure of galacturonic acid was preliminarily constructed using the Chemdraw10.0 program in the Chemoffice package. Then, the objective molecule structure was imported into the HyperChem 7.0 program, the molecular structure was preliminarily optimized by the molecular force field of the MM+ method, and then the structure was subsequently optimized using the semi-empirical MNDO method to calculate the net charge distribution of each atom in the molecule. Calculated results were used for the following drying mechanism analysis.

### 2.6. Structural Characterization of SBP, Ca^2+^-SBP, and Cu^2+^-SBP Using FTIR

To further disclose the possible drying mechanism of the crosslinking method, a scanning test was performed for three samples of SBP, Ca^2+^-SBP, and Cu^2+^-SBP using a Fourier Transform Infrared Spectrometer (FTIR, German BRUKER Company, Karlsruhe, Germany, VECTOR-33). So, the difference among functional groups can be observed. A KBr pellet as a blank sample was put into a sample cell, and a reference background spectrum was obtained for 3 samples. Then, 3 samples were put in a mortar and ground into powder. Then, a certain KBr was added to 3 cells, and 3 pellets were made. Then, 3 pellets were placed in an FTIR. The scanning wavelength ranged from 400~4000 cm^−1^ using an FTIR with a resolution of 4 cm^−1^.

### 2.7. Structural Characterization of SBP, Ca^2+^-SBP, and Cu^2+^-SBP Using XRD

To further analyze the physical property and chemical structure of three samples, like SBP, Ca^2+^-SBP, and Cu^2+^-SBP; to compare the difference in crystallization degree; and to clarify the possible mechanism of crosslinking reaction improving drying energy consumption and velocity, an XRD analysis was performed. An X-ray powder diffractometer (XRD, German BRUKER Company, Karlsruhe, Germany, D/max2200VPC) was used with a tube pressure of 50 kV, a tube current of 40 mA, a scanning angle *(2θ)* range of 5°to 50°, and a scanning speed of 38.5 s/step.

### 2.8. Structural Characterization of SBP, Ca^2+^-SBP, and Cu^2+^-SBP Using SEM

Three samples, like SBP, Ca^2+^-SBP, and Cu^2+^-SBP, were further analyzed to determine their surface morphology. A scanning electron microscope (SEM, German BRUKER Company, Karlsruhe, Germany, ZEISS EVO18) was used with a tungsten lamp. The three samples needed to be gold-plated under vacuum conditions before analysis.

## 3. Results and Discussion

### 3.1. Effects of Different Crosslinking Metal Ions on SBP Dryingness

**Figure 2:**
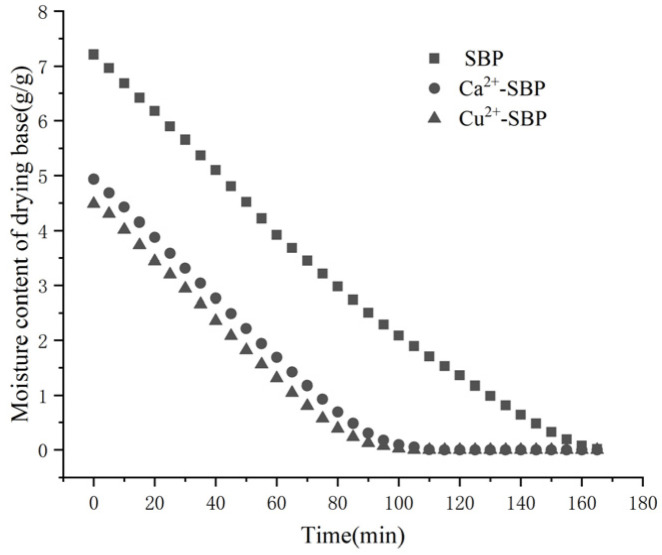
Moisture content of drying base of SBP, Ca^2+^-SBP, and Cu^2+^-SBP.

According to Figure 2, the dry basis moisture contents of Ca^2+^-SBP and Cu^2+^-SBP were 4.93 and 4.48 g/g, respectively. Based on the temperature of 60 °C, the drying times were 110 and 100 min, respectively. The dry basis moisture content of untreated SBP was 7.21 g/g, and the drying time was 165 min. Compared with SBP, the dry basis moisture content of the crosslinking SBP was reduced by 30~40%, which significantly shortened the drying time. Although the dry basis moisture contents and drying times of Ca^2+^-SBP and Cu^2+^-SBP showed no significant difference, the level of addition in feed varied greatly, with the addition of calcium being about 100 to 400 times that of copper. Copper has potential toxicity, and the maximum limit for piglets is 125 mg/kg [13]. Calcium is the most abundant mineral element in the animal body and is known as the “element of life.” Generally speaking, the calcium obtained from natural formulated feed is not enough for livestock and poultry according to nutritional needs, and an additional calcium source needs to be supplemented [14]. For the large-scale drying treatment of sugar beet pulp and as a feed additive, Ca^2+^-SBP is more advantageous.

**Figure 3:**
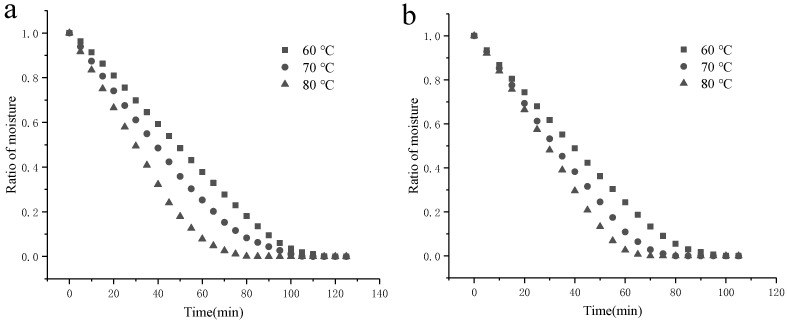
Hot-air drying curves of Ca^2+^-SBP (**a**) and Cu^2+^-SBP (**b**) at different hot-air temperatures of 60 °C, 70 °C, and 80 °C.

**Figure 4:**
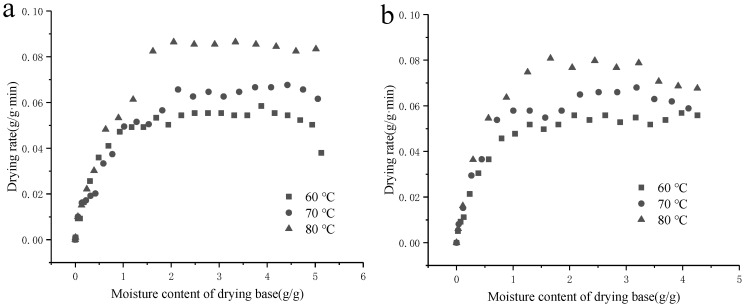
Hot-air drying rate of Ca^2+^-SBP (**a**) and Cu^2+^-SBP (**b**) at different hot-air temperatures of 60 °C, 70 °C, and 80 °C.

Under different hot-air temperatures (60 °C, 70 °C, and 80 °C), the moisture ratio and drying rate curves of SBP, Ca^2+^-SBP(a), and Cu^2+^-SBP(b) are shown in Figure 3 and Figure 4.

As shown in Figure 3 and Figure 4, the drying rate of Ca^2+^-SBP, Cu^2+^-SBP, CDSBP, and GDSBP all increased with the increase in the hot-air temperature. At a lower temperature of 60 °C, the surface moisture of SBP evaporates slowly, and the internal diffusion and migration forces are small, resulting in a low drying rate of SBP and a long drying time. When the temperature increases, the cell walls of the SBP break, and micropores disappear and turn into macropores, in which humidity is retained with a lower interaction force. Thus, the desorption and diffusion of content through the crust toward the outer surface become easier [15], thereby accelerating the drying of SBP.

### 3.2. Effects of Metal Ion Concentrations, Operating Temperatures, and Solution pH on the Drying Process

#### 3.2.1. Effect of Ca^2+^ or Cu^2+^ Concentrations on the Drying Process of SBP

**Figure 5:**
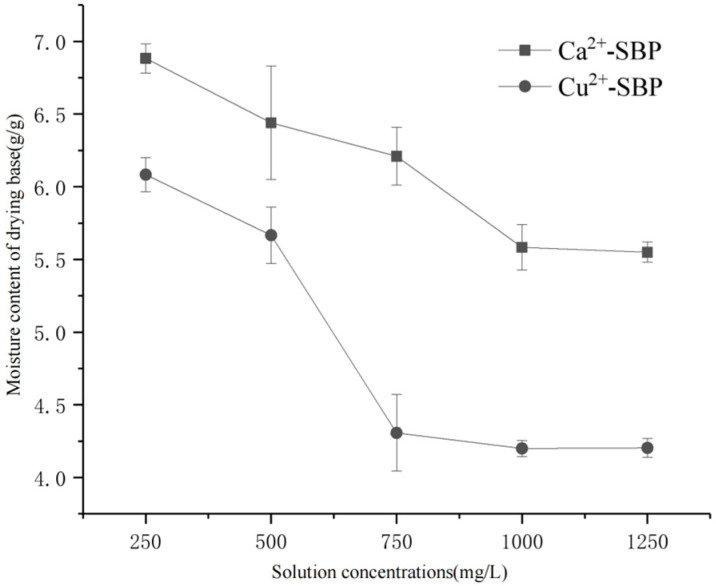
Moisture content of drying base of SBP at different cationic solution concentrations.

To discuss the effect of metal ion concentration on the moisture content of SBP through a crosslinking reaction, according to Section 2.3.1, a CaCl_2_ solution and a CuCl_2_ solution of 250, 500, 750, 1000, and 1250 mg/L were prepared for later use, respectively. Results are shown in Figure 5.

According to Figure 5, with the increase in Ca^2+^ or Cu^2+^ concentration in the range of 250~250 mg/L, the moisture content of drying base decreased when Ca^2+^-SBP and Cu^2+^-SBP were dried to a constant weight. In addition, the moisture content decreased in a sharp trend first and then in a gentle trend, indicating a saturation adsorption of Ca^2+^ or Cu^2+^ onto SBP.

At a concentration of 1000 mg/L and 1250 mg/L for Ca^2+^, the moisture contents are 5.69 g/g and 5.64 g/g, respectively. At this time, the drying velocity is the fastest, with the shortest drying time of 150 min. The reason for this may be that the adsorption of Ca^2+^ onto SBP reached saturation, and then the moisture content of the dry base becomes constant for Ca^2+^-SBP. So, in the following experiment, a 1000 mg/L concentration of Ca^2+^ is selected. However, at a 750 mg/L Cu^2+^ concentration, the moisture content of the dry base for Cu^2+^-SBP is 4.3 g/g, and the drying time to a constant weight is 135 min. At a Cu^2+^ concentration of 1000 or 1250 mg/L, the moisture content of the dry base for Cu^2+^-SBP is close to that of 750 mg/L. So, a 750 mg/L concentration of Cu^2+^ was selected for the latter experiment. Unlike Ca^2+^, Cu^2+^ has a better binding to SBP. So, the drying time of Cu^2+^-SBP is shorter than that of Ca^2+^-SBP.

#### 3.2.2. Effect of Operating Temperatures on the Drying Process of SBP

**Figure 6:**
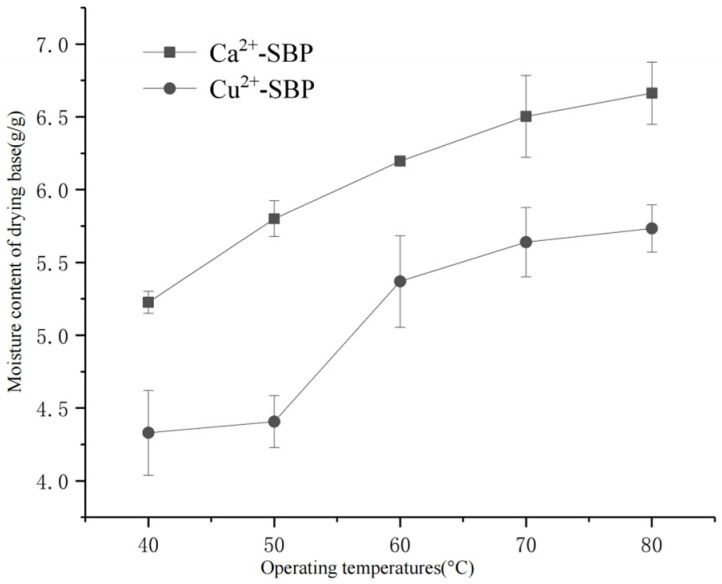
Moisture content of drying base of Ca^2+^-SBP and Cu^2+^-SBP at different operating temperatures.

The effect of crosslinking temperature on the drying of SBP was performed following Section 2.3.2, and the results are shown in Figure 6. Results showed that in the drying process of SBP, an operating temperature within a range of 40~80 °C had an effect on the moisture content of the dry base and drying time, as shown in Figure 6.

According to Figure 6, with an increase in operating temperature, Ca^2+^-SBP and Cu^2+^-SBP exhibit an increasing moisture content at the final drying time. At a bath or operating temperatures of 40 °C, Ca^2+^-SBP has the lowest moisture content of 5.23 g/g and the shortest drying time of 135 min. At 80 °C, the moisture content and the drying time of Ca^2+^-SBP were 7.32 g/g and 195 min, respectively. At a bath temperature of 40 °C or 50 °C, Cu^2+^-SBP has the lowest moisture content of 4.33 g/g and 4.4 g/g, respectively. With increasing temperature, the moisture content increases, and the drying time becomes longer. Possible reasons ascribed to an increasing temperature have a negative effect on the Ca^2+^ adsorption onto SBP and on the swelling and water-holding ability of SBP. In the future, more experiments will be needed to test the effect of Ca^2+^ or Cu^2+^ adsorption on the swelling and water-holding ability.

#### 3.2.3. Effect of Solution pH on the Drying Process of SBP

**Figure 7:**
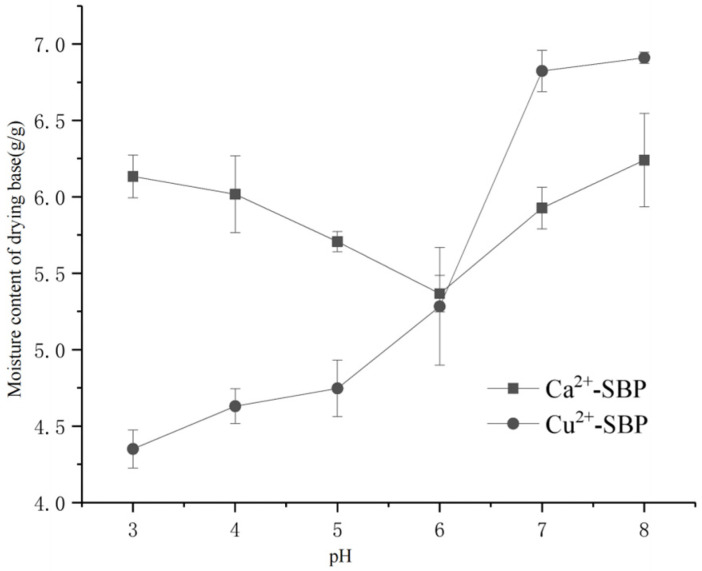
Moisture content of drying base of Ca^2+^ -SBP and Cu^2+^ -SBP at different pH.

According to Section 2.3.3, the effect of different solution pH, including pH 3.0, 4.0, 5.0, 6.0, 7.0, and 8.0, on the moisture content and drying time of Ca^2+^-SBP and Cu^2+^-SBP was determined, as shown in Figure 7. According to Figure 7, the moisture content decreased first and then increased with increasing pH value. At pH 6.0, the lowest moisture content of Ca^2+^-SBP is 5.36 g/g with a final drying time of 135~150 min. At pH 8.0, the moisture content of Ca^2+^-SBP is 6.24 g/g, greater than that at pH 6.0. At a low pH value within pH 3.0~pH 6.0, Cu^2+^ competes with H^+^ to bind with hydroxyl groups, leading to a lower Ca^2+^ adsorption capacity than at pH 7.0 or 8.0. So, the moisture content of Ca^2+^-SBP at pH 3.0~6.0 is lower than that at pH 7.0 or 8.0.

Within the range of 3.0~8.0, Cu^2+^-SBP shows an increasing trend for its moisture content on a dry basis, with an increase in pH value. So, at pH 3.0, Cu^2+^-SBP has the lowest moisture content. The cause of this phenomenon may be explained by the surface charge on SBP. The hydrolysis of CuCl_2_ leads to the binding of Cu^2+^ with OH^-^, and Cu^2+^ binds with carboxyl groups of D-Galacturonic acid at the same time. So, the hydrolysis weakened the binding of Cu^2+^ with carboxyl groups of D-Galacturonic acid, leading to a higher water-holding capacity and a higher moisture content of the dry base.

Since wet SBP is usually in a solution with a pH around 7, Ca^2+^ is more practical for strengthening the drying process due to its crosslinking reaction than Cu^2+^. According to our practice and literature reports, energy decreases at least by 10% [16].

### 3.3. Fitting to Drying Mathematical Models

In order to fit the experimental values, different mathematical drying models are listed in Table 1 from different literature sources.

Based on the literature models in Table 1 and the SBP-drying kinetic data from different processing methods and different temperatures, different mathematical drying models were established. In Table 2, the results showed that the Midilli–Kucuk model was the best one to fit with the experimental data, and an R^2^ of 0.9971~0.9992 was the closest to 1. Therefore, this model was selected to describe the hot-air drying process of SBP under different treatment methods.

**Figure 8:**
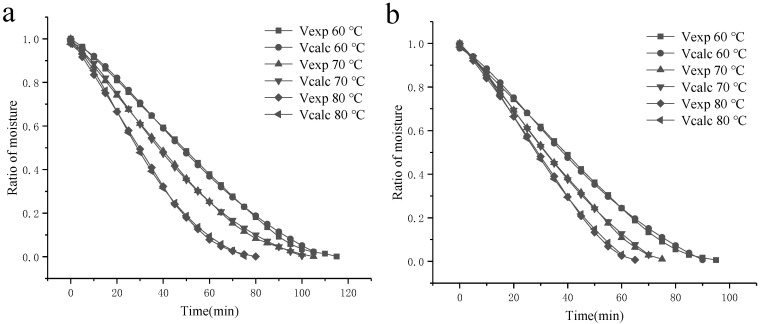
Comparison of the moisture ratio of Ca^2+^-SBP (**a**) and Cu^2+^-SBP (**b**) resulting from experimental measurements with those calculated by the proposed model.

As shown in Figure 8, by comparing the results of experimental data of the moisture ratio with the results calculated based on the proposed model, good agreement was observed across the entire study temperature range.

### 3.4. Characterization of SBP, Ca^2+^-SBP, and Cu^2+^-SBP Using FTIR, XRD, and SEM

**Figure 9:**
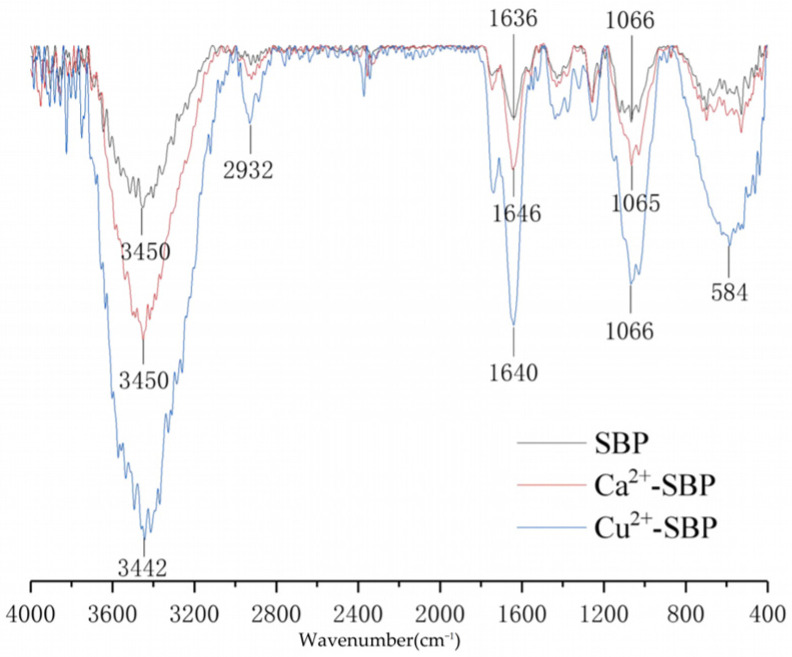
FTIR spectra of SBP, Ca^2+^-SBP, and Cu^2+^-SBP.

According to Section 2.6, the FTIR spectra of SBP, Ca^2+^-SBP, and Cu^2+^-SBP are shown in Figure 9. For SBP, three characteristic absorbance peaks of 1066, 1636, and 3450 cm^−1^ are assigned as three functional groups of C-O-C, COO^-^, and O-H, respectively. Among them, the peak at 1066 cm^−1^ is from the stretching vibration of C-O-C as a pyranose skeleton, a typical characteristic of cellulose. Unlike SBP, the absorption peak strength weakened to a great degree for the three peaks of 3450, 1646, and 1065 cm^−1^ from Ca^2+^-SBP. In comparison to SBP and Ca^2+^-SBP, 5 absorbance peaks of 3442, 2932, 1640, 1066, and 584 cm^−1^ from Cu^2+^-SBP weakened in their peak strength to a greater degree. In addition, the absorbance peak around 3200~3700 cm^−1^ is from O-H and COOH, and the weakening indicates the success of the crosslinking reaction. So, the water-holding capacity decreased to a certain degree.

**Figure 10:**
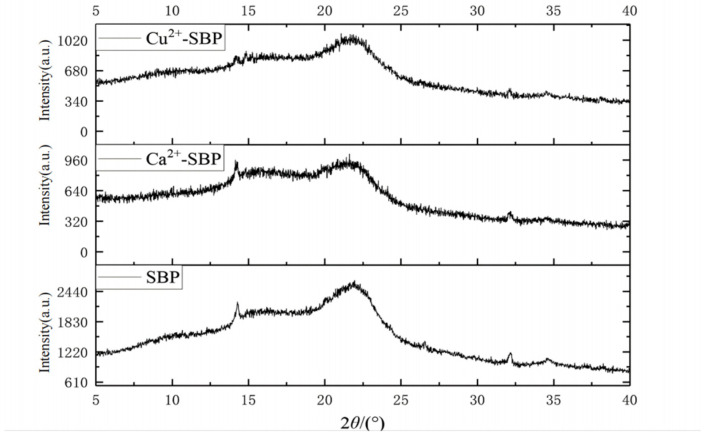
XRD patterns of SBP, Ca^2+^-SBP, and Cu^2+^-SBP.

According to the method described in Section 2.7, the X-ray diffraction patterns of SBP, Ca^2+^-SBP, and Cu^2+^-SBP are shown in Figure 10. The pattern reveals distinct diffraction peaks at 15.8° and 21.8°, with the 21.8° peak being particularly prominent. These peaks correspond to characteristic cellulose structures, consistent with a previous study [25]. In comparison, both Ca^2+^-SBP and Cu^2+^-SBP exhibit significantly weaker diffraction peaks at 21.8°. This phenomenon may result from crosslinking interactions between pectin in SBP and metal cations, which reduce crystal volume and decrease crystallinity. So, some hydrophilic groups, such as -OH, decreased, and as a result, the water-holding capacity of SBP decreased through a crosslinking reaction.

**Figure 11:**
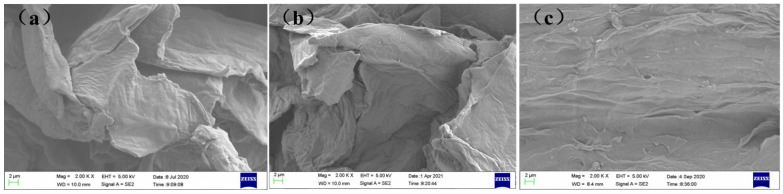
SEM images of SBP (**a**), Ca^2+^-SBP (**b**), and Cu^2+^-SBP (**c**).

To further differentiate the morphology difference of SBP, Ca^2+^-SBP, and Cu^2+^-SBP, SEM was used to analyze their micro-structure difference according to Section 2.8. The SEM images of three samples with a 2000 magnification are shown in Figure 11. According to Figure 11, unlike SBP, Ca^2+^-SBP and Cu^2+^-SBP have better firmness and smaller pore diameter. In addition, SBP has a smoother surface than Ca^2+^-SBP and Cu^2+^-SBP; the latter two have some small bumps, and the crosslinking reaction might be the cause of the bumps. In addition, unlike SBP and Ca^2+^-SBP, Cu^2+^-SBP has a flattening surface morphology in the macro-lever other than in the detail-lever, since SBP and Ca^2+^-SBP have macroscopic curls. The flattening surface morphology may be attributed to the superior crosslinking ability of Cu^2+^ [26,27].

### 3.5. Drying Mechanism

#### 3.5.1. Electronegativity Calculation

**Figure 12:**
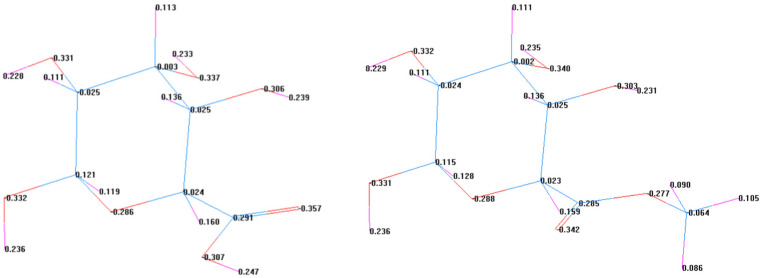
Theoretical electronegativity charge analysis of galacturonic acid and methyl galacturonic acid. Blue, red, and pink represent carbon, oxygen, and hydrogen atoms, respectively.

Figure 12 shows the theoretical calculation results regarding the net charge of each atom in galacturonic acid or in galacturonic acid with methyl. As shown in Figure 12, all oxygen atoms are negatively charged. Generally, the more negatively charged atoms are, the stronger the nucleophilicity is, and the stronger the ability to form complexes by bonding with metals is. In the molecules of galacturonic acid and methyl galacturonic acid, the oxygen atoms of carboxyl groups have the most negative charge (−0.357 and −0.342, respectively). Therefore, when galacturonic acid and methyl galacturonic acid crosslink with calcium ions, the carboxyl oxygen atoms react with calcium ions first and have a strong ability to form complexes. Generally speaking, the crosslinking reaction benefits the drying process, and the most probable reaction position is the carboxyl group from sugar beet pectin [28].

#### 3.5.2. Chemical Reaction Process and Possible Dryness–Strengthening Mechanism

**Figure 13:**
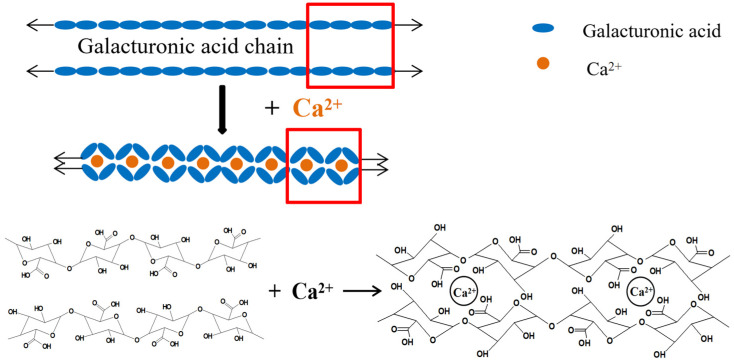
Crosslinking process of galacturonic acid chains with Ca^2+^. The red boxes indicate the structural change before and after the galacturonic acid chains crosslink with Ca^2+^.

As shown in Figure 13, galacturonic acid contains many active groups, such as the carboxyl group and hydroxyl groups. When metal cations interact with the galacturonic acid chain, the carboxyl groups in the two adjacent galacturonic acid chains exhibit a synergistic crosslinking effect with metal cations, reducing water binding in SBP and thereby forming denser tissues, which saves drying time and energy. So, the crosslinking process improves SBP-pressing performance [24], just like Ca^2+^, as an example.

For the unmethylated polygalacturonic acid molecule, calcium ions replace hydrogens of the carboxyl group of adjacent molecules to form a spatial network structure and a stable state. Under appropriate conditions, polygalacturonic acid is partially methyl-esterified (methanolization; that is, the formation of methanol esters), the main component of which is partially methylized α-(1, 4)-D-polygalacturonic acid. In low-methoxy pectin, due to its low DE value and relatively great number of COO- groups, calcium ions make two adjacent low-methoxy pectin molecular chains close to each other through electrostatic interaction, forming an “egg box” model structure [29], as shown in Figure 13.

In addition, according to the theory of hard and soft acids and bases (HSAB), for the reactions of partially methylated α-(1, 4)-D-polygalacturonic acid with calcium ions, Ca^2+^ is a hard acid, which is bound to the hard base of COO- in polygalacturonic acid. When the hard acid and hard base react, the product has high stability and a fast reaction speed [30].

## 4. Conclusions

Based on the consideration of environmental protection, energy saving, and harmless high-value application of bio-adsorbent, this study used SBP to crosslink with Ca^2+^ or Cu^2+^. Under experimental conditions, wet SBP with 2 g of dry base reacts to Ca^2+^ under optimized conditions of 1000 mg/L Ca^2+^ solution at pH 6.0 and 40 °C for 135 min, with a moisture content of 5.23 g/g as a water-holding index. Compared with SBP, the moisture content of the crosslinking SBP on a dry basis was reduced by 30~40%. Ca^2+^ or Cu^2+^ can crosslink to the galacturonic acid of pectin and form an “egg box” model through hydroxyl or carboxyl’s negative charge or other weak interaction, as testified by a combination analysis of FTIR, SEM, and XRD. Mathematical drying models under different SBP treatment methods and different hot-air temperatures were established. Among them, the Midilli–Kucuk model fits well and is in good agreement with the experimental values.

The dryness-strengthening and value-added idea for drying SBP through a Ca^2+^-crosslinking method is practical. The SBP structure becomes more compact with a decreasing holding-water capacity and a lower moisture content after the crosslinking reaction, thereby significantly reducing the water content of SBP and reducing the required drying time. Meanwhile, Ca^2+^-SBP can be directly used as a feed additive to increase the nutritional value of feed. Moreover, Ca^2+^-SBP can be used as a biosorbent to treat wastewater, with advantages such as no subsequent regeneration treatment or harmful treatment problems. This study laid a foundation for the large-scale energy-saving drying of other agricultural product processing by-products, such as bananas and pineapples, which have good prospects for industrial application.

In the future, to realize the industrialization and valorization of SBP, we can conduct further research on the effect of pilot processing conditions on SBP nutrition and confirm that Ca^2+^-SBP or Cu^2+^-SBP has a better feed value, although it is commonly reported [11,15,16]. Although CuSO_4_ is a feed additive without toxicity in a certain dose, more work can be performed to test Cu^2+^-SBP’s toxicity as a feed additive.

## Figures and Tables

**Table 1:** Thin-layer drying models.

Model Types	Model Equation	References
Henderson and Pabis	MR = a·exp (−k·t)	[17]
Exponential	MR = exp (−k·t)	[18]
Logarithmic	MR = c + a·exp (−k·t)	[19]
Parabolic	MR = a + b·t + c·t^2^	[20]
Wang and sigh	MR = 1 + b·t + c·t^2^	[21]
Page	MR = exp (−k·t^n^)	[22]
Weibull	MR = exp (−(t/α)^β^)	[23]
Midilli–Kucuk model	MR = a·exp (−kt^n^) + bt	[24]

**Table 2:** Coefficients of Midilli–Kucuk exponential model fitted with drying data from different samples.

Samples	Constants	60 °C	70 °C	80 °C
Ca^2+^-SBP	a	0.9841	0.9795	0.9755
	k	0.0016	0.0033	0.0037
	n	1.5100	1.4390	1.5290
	b	−0.0012	−0.0008	−0.0008
	R^2^	0.9987	0.9989	0.9984
Cu^2+^-SBP	a	0.9775	0.9899	0.9825
	k	0.0031	0.0049	0.0034
	n	1.4240	1.3600	1.5200
	b	−0.0016	−0.0025	−0.0025
	R^2^	0.9983	0.9992	0.9986

## Data Availability

The original contributions presented in the study are included in the article; further inquiries can be directed to the corresponding author.
